# Hydrogen Sulfide Suppresses Outward Rectifier Potassium Currents in Human Pluripotent Stem Cell-Derived Cardiomyocytes

**DOI:** 10.1371/journal.pone.0050641

**Published:** 2012-11-30

**Authors:** Heming Wei, Guangqin Zhang, Suhua Qiu, Jun Lu, Jingwei Sheng, Manasi, Grace Tan, Philip Wong, Shu Uin Gan, Winston Shim

**Affiliations:** 1 Research and Development Unit, National Heart Centre Singapore, Singapore, Singapore; 2 Graduate Medical School, DUKE-National University of Singapore, Singapore, Singapore; 3 Department of Surgery, Yong Loo Lin School of Medicine, National University of Singapore, Singapore, Singapore; Indian Institute of Toxicology Reserach, India

## Abstract

**Aim:**

Hydrogen sulfide (H_2_S) is a promising cardioprotective agent and a potential modulator of cardiac ion currents. Yet its cardiac effects on humans are poorly understood due to lack of functional cardiomyocytes. This study investigates electrophysiological responses of human pluripotent stem cells (hPSCs) derived cardiomyocytes towards H_2_S.

**Methods and Results:**

Cardiomyocytes of ventricular, atrial and nodal subtypes differentiated from H9 embryonic stem cells (hESCs) and human induced pluripotent stem cells (hiPSCs) were electrophysiologically characterized. The effect of NaHS, a donor of H_2_S, on action potential (AP), outward rectifier potassium currents (*I*_Ks_ and *I*_Kr_), L-type Ca^2+^ currents (*I*_CaL_) and hyperpolarization-activated inward current (*I*_f_) were determined by patch-clamp electrophysiology and confocal calcium imaging. In a concentration-dependent manner, NaHS (100 to 300 µM) consistently altered the action potential properties including prolonging action potential duration (APD) and slowing down contracting rates of ventricular-and atrial-like cardiomyocytes derived from both hESCs and hiPSCs. Moreover, inhibitions of slow and rapid *I*_K_ (*I*_Ks_ and *I*_Kr_), *I*_CaL_ and *I*_f_ were found in NaHS treated cardiomyocytes and it could collectively contribute to the remodeling of AP properties.

**Conclusions:**

This is the first demonstration of effects of H_2_S on cardiac electrophysiology of human ventricular-like, atrial-like and nodal-like cardiomyocytes. It reaffirmed the inhibitory effect of H_2_S on *I*_CaL_ and revealed additional novel inhibitory effects on *I*_f_, *I*_Ks_ and *I*_Kr_ currents in human cardiomyocytes.

## Introduction

Safety and efficacy evaluation of pharmaceuticals for cardiac indications are hindered due to a shortage of suitable human *in vitro* cellular models. Such limitation has in part precipitated in market withdrawal of several cardiac as well as non-cardiac drugs recently [1], [2]. Current available cardiotoxicity screening platform that relying on animal-derived cardiomyocytes or cell lines ectopically expressing ion channels are not ideal due to species difference in the former and inadequate ion channel interactions in the latter [3]. Human pluripotent stem cell (hPSC) including human embryonic stem cells (hESC) and human induced pluripotent stem cells (hiPSCs) are capable of cardiomyogenesis [4], [5]. hESC- and hiPSC-derived cardiomyocytes (hESC-CMs and hiPSC-CMs) present an unprecedented window for an expedited evaluation of chemical entities for known cardiac toxicity or hitherto unknown cardiac implications. In contrast to hESCs, hiPSCs are capable of giving rise to a renewable source of cardiomyocytes from individual patients. These patient-derived cardiomyocytes offer an immensely valuable resource in evaluating individual-specific responses towards pharmaceutical agents especially anti-arrhythmics that have a narrow therapeutic index that are often compounded by idiosyncratic patient response.

Hydrogen sulfide (H_2_S) is a recently identified physiological gaseous molecule associated with cardiovascular benefits such as vasodilation and cardiac protection [6]. It is known to affect multiple ion channels that could have implication in cardiac arrhythmia [7]. However, its influence on electrical remodeling of human cardiomyocytes is yet to be understood and its effect on individual ventricular, atrial and nodal cardiomyocyte subtypes has not been explored.

**Figure 1:**
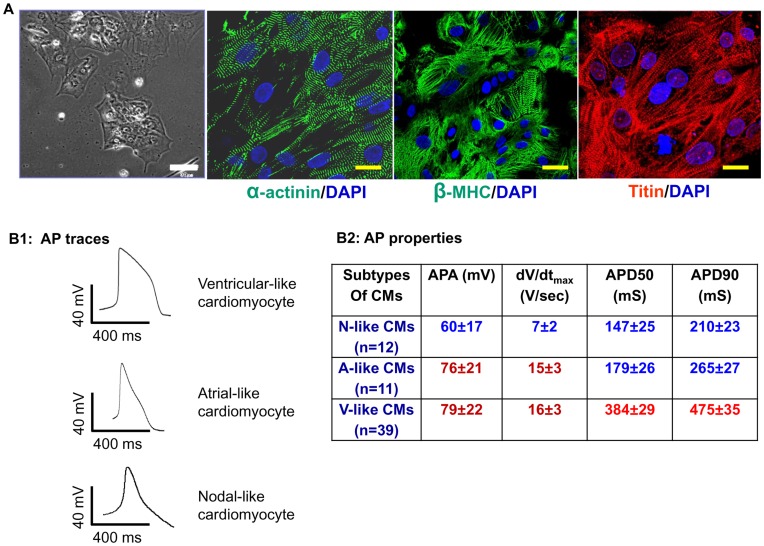
Generation and characterization of cardiomyocytes differentiated from H9 hESCs. A: Light microscopy and immunocytochemistry image of H9 hESC-CMs dissociated from contracting EBs. Scale bar: 100 µm. Fluorescent images of H9 hESC-CMs stained with antibodies against α-actinin, β-MHC and Titin showed typical cardiac sarcomeres. Scale bar: 30 µm. B1∼2: Action potential trace of three subtypes of cardiomyocytes derived from H9 hESCs and corresponding specific action potential properties. APA: action potential amplitude. dV/dt_max_: maximal rate of depolarization or maximal upstroke velocity. APD50: action potential duration at 50%. APD90: action potential duration at 90%.

**Figure 2:**
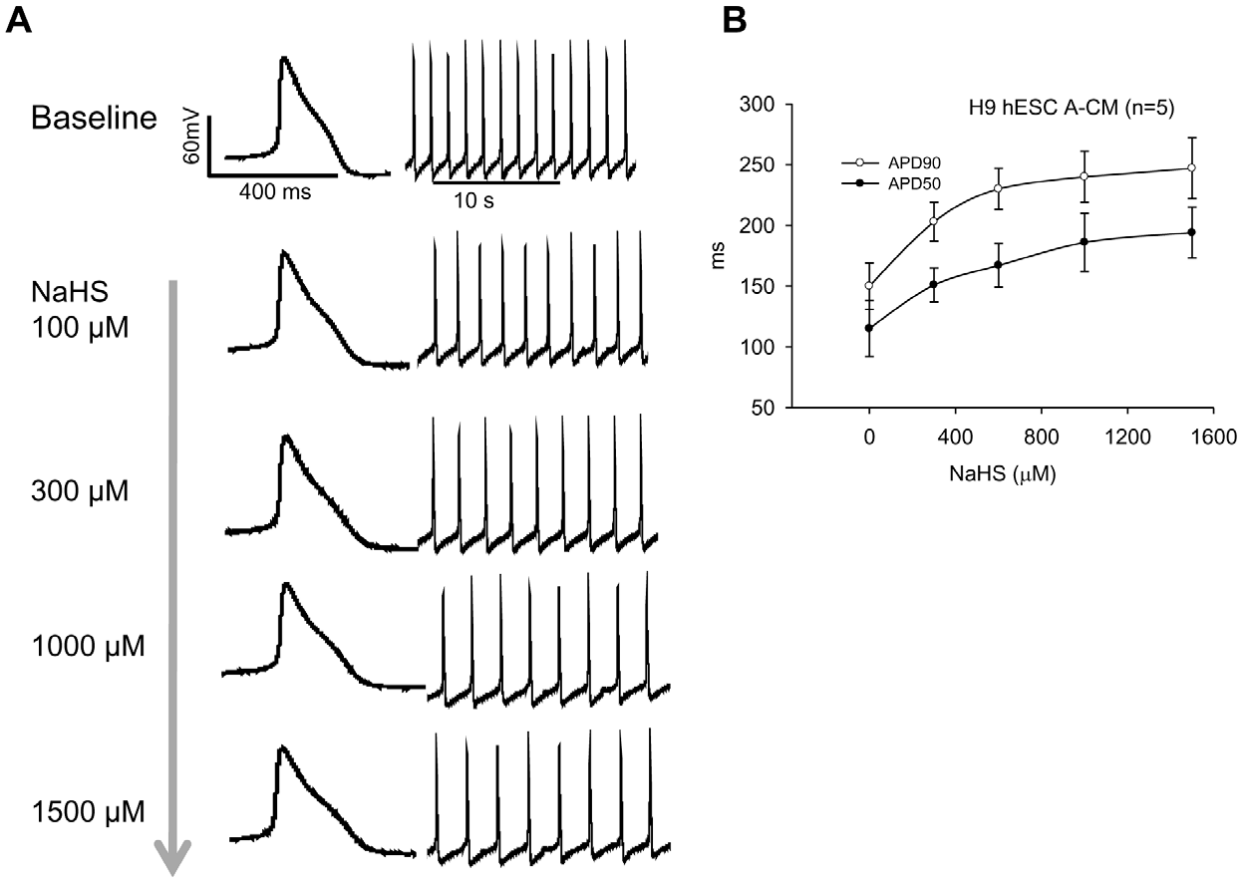
Dose-dependent effect of H_2_S on H9 hESC-CMs. A: A H9 hESC-derived atrial-like cardiomyocyte concentration-dependently responded to an increasing dose (100∼1500 µM) of NaHS presented as prolongation of action potential duration (APD50 and ADP90) and slowing of contraction rates. B: The corresponding IC_50_ curve.

In this study, we demonstrated that H_2_S altered the action potential (AP) properties of hESC- and hiPSC-derived ventricular (V)-, atrial (A)-like CMs. H_2_S not only blocked L-type Ca^2+^ current (*I*_CaL_), but also showed inhibitory effects on the slow and rapid outward rectifier K^+^ currents (*I*_Ks_ and *I*_Kr_).

## Methods

### Generation of Human Embryonic Stem Cell-derived Cardiomyocytes

H9 hESCs (WiCell, Madison, USA) were maintained on mouse embryonic fibroblasts feeder (Millipore, USA) in hESC medium (80% Knockout Dulbecco’s Modified Eagle Medium or DMEM, 20% Serum replacement, 1% non-essential amino acid, 1 mM L-glutamine, 0.1 mM beta-mercaptoethanol and 4 ng/ml bFGF). Unless specified, all culture reagents were from Invitrogen. Embryoid bodies (EBs) were generated after mechanical dissection of hESCs before being maintained in suspension culture in a cardiomyogenic medium which contains DMEM High Glucose 485 ml, L-GLutamine 5 ml, NEAA 5 ml, Selenium Transferrin 5 ml (Sigma) and 2-mercaptoethanol 3.5 ul supplemented with 5 µM SB 203580 (Sigma), a specific p38-MAPK inhibitor in low adherent 6-well plates (Corning, USA) [8]. Subsequently, contracting EB aggregates (emerged from Day 15 onwards) were plated on 0.1% gelatin coated dishes and maintained in DMEM containing 2% FBS. On day 21, the contracting outgrowth of cardiomyocytes were mechanically dissected out and dissociated in Collagenase B (Roche) [9] into small cell clusters (containing 10–30 cells) and continually cultured in DMEM supplemented with 2% FBS for 2 weeks before examination.

### Characterization of Human Pluripotent Stem Cell-derived Cardiomyocytes

Human iPSC-derived cardiomyocytes (hiPSC-CMs) were acquired from Cell Dynamic International (CDI, Wisconsin, USA) and they have been well characterized [10]. All cardiomyocytes used in this study were 5 weeks post cardiac differentiation. For structural characterization, cardiomyocytes were seeded on glass coverslips and immunocytochemically stained for sarcomeric cardiac markers using antibodies against cardiac sarcomeric α-actinin (clone EA-35. Sigma), β-myosin heavy chain or β-MHC (Alexis Biochemicals, FL, USA) followed by Alexa Fluo® 488 goat anti-rabbit IgG (Invitrogen, CA, USA); and cardiac Titin (1∶10) (Sigma-Alrich, MO, USA) followed by Alexa Fluo® 555 donkey anti-rabbit IgG. For electrophysiological characterization, H9 hESC-CMs and hiPSC-CMs were identified with spontaneous action potential (AP) recorded by Patch-Clamp technology later described. Ventricular (V)-, atrial (A) - and nodal (N)-like subtypes of cardiomyocytes were determined by their characteristic AP properties [11].

**Figure 3:**
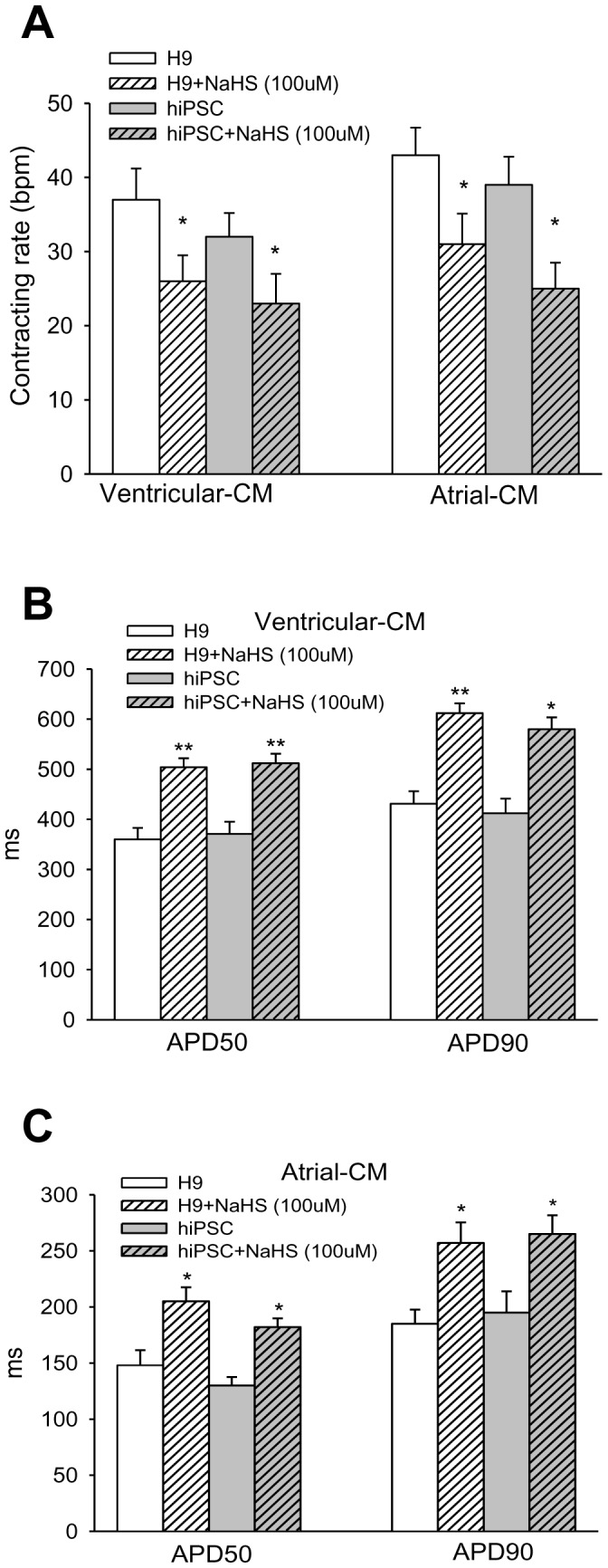
Action potential response of hESC- and hiPSC-CMs towards H_2_S. A∼B: Responses of action potential traces of H9 hESC- and hiPSC-derived V- and A-like CMs towards H_2_S. Changes of the AP properties of different subtypes of cardiomyocytes before and after NaHS (100 µM) exposure were analyzed. C: Beating rates in H9 hESC- and hiPSC-derived V- and A-like CMs. D∼E: Action potential duration (APD) in the V-like CMs (D) and A-like CMs (E). *P<0.05; **p<0.01 (NaHS treated vs. baseline).

**Figure 4:**
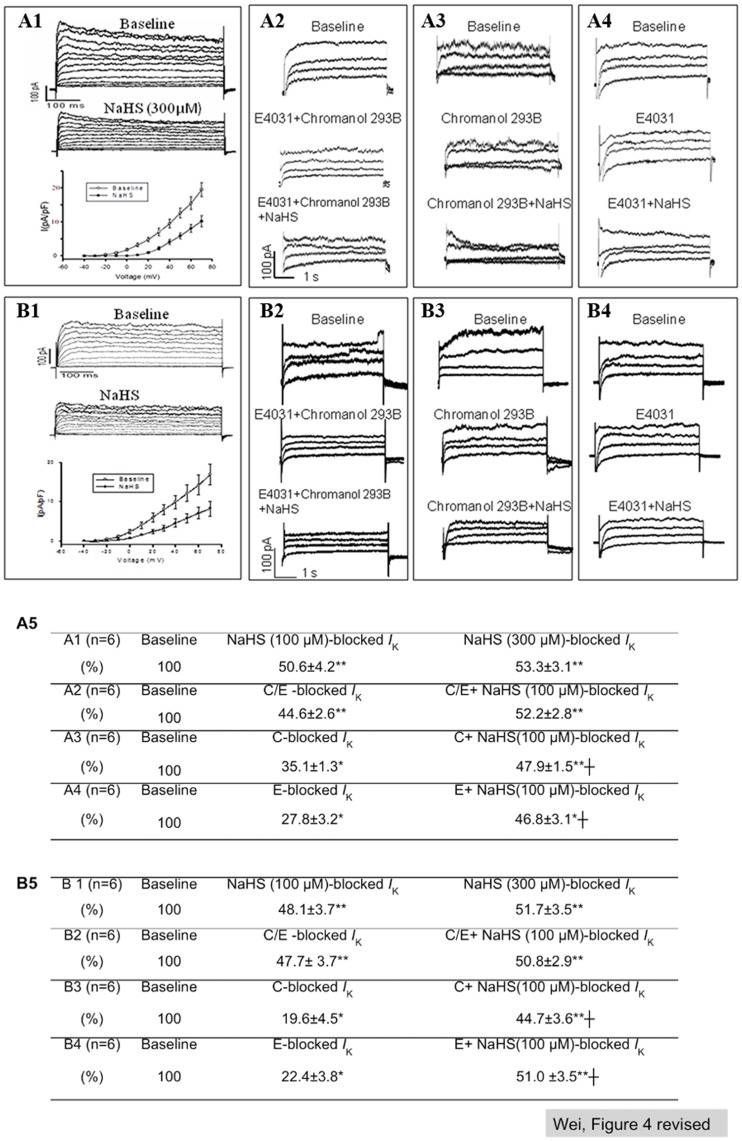
I_K_ response of hESC- and hiPSC-CMs towards H_2_S. A, B: I_K_ recorded on V-CMs derived from H9 hESCs (A) and hiPSCs (B) and A1, B1: I_K_ recorded before and after NaHS (300 µM) treatment. A2, B2: I_K_ recorded at baseline, with pre-treatment with a mixture of 5 µM E-4031 and 5 µM Chromanol 293B, and with subsequently treatment with NaHS (100 µM). A3, B3: I_K_ recorded at baseline, with pre-treatment with 5 µM Chromanol 293B, and with subsequently treatment with NaHS (100 µM). A4, B4: I_K_ recorded at baseline, with pre-treatment with 5 µM E-4031, and with subsequently treatment with NaHS (100 µM). A5, B5: Quantification of I_K_ response of hESC-CMs (A5) and hiPSC-CMs (B5) towards Chromanol 293B, E-4031 and NaHS observed in A1∼A4 and B1∼B4, respectively. Relative quantity (% of baseline I_K_) of Chromanol 293B-, E-4031- and NaHS-sensitive I_K_ were calculated by subtracting post-treatment I_K_ from baseline. *P<0.05; **p<0.01 (NaHS or I_K_ blocker(s) treated vs. baseline). ┼p<0.05 (NaHS treated vs. I_K_ blocker treated). C/E: Chromanol 293B+E-4031; C: Chromanol 293B; E: E-4031. Number of repeats (n) = 6.

### Evaluation of the Effect of H_2_S on hESC- and hiPSC-CMs

Freshly prepared NaHS stock solution (1000 mM) was added to cardiomyocytes using a micro-perfusion system. For the concentration curve, NaHS was sequentially added to the cells from low to high concentrations ranging from 100 to 1500 µM.

### Patch-clamp Electrophysiology

Cardiomyocytes seeded on 3.5 cm diameter petri dishes were transferred to a recording chamber mounted on the stage of an inverted microscope (TE2000-S, Nikon, Tokyo, Japan). Whole-cell AP and ion currents were recorded with a Patch-Clamp amplifier (Axon 200B, Axon Instruments, Foster City, CA, USA). Patch pipettes were fabricated with a Sutter P-97 horizontal puller (Sutter Instrument, Novato, CA) and had a resistance of 2–4 M*Ω* when filled with the internal solution. In experiments, 70–90% series resistance was compensated. Currents and voltage protocol generation, data acquisition and analysis, were performed using Clampex and Clampfit software (version 10.0, Axon Instruments). Except Ca^2+^ current measurement, all experiments were performed at 37°C.

#### Measurement of action potential

V-, A- and N-like CMs were identified by action potential (AP) patterns recorded by whole cell patch-clamp configuration in normal Tyrode’s solution [(in mM): NaCl 140, KCl 5.4, CaCl_2_ 1.8, MgCl_2_ 1, glucose 10, HEPES10, pH 7.4 with NaOH] in current-clamp mode. From H9 hESC-CMs, the AP homogeneity of the subpopulation within each CM cluster (10 to 30 CMs) was determined by testing all “patchable” cardiomyocytes (over 70%) within the cluster.

**Figure 5:**
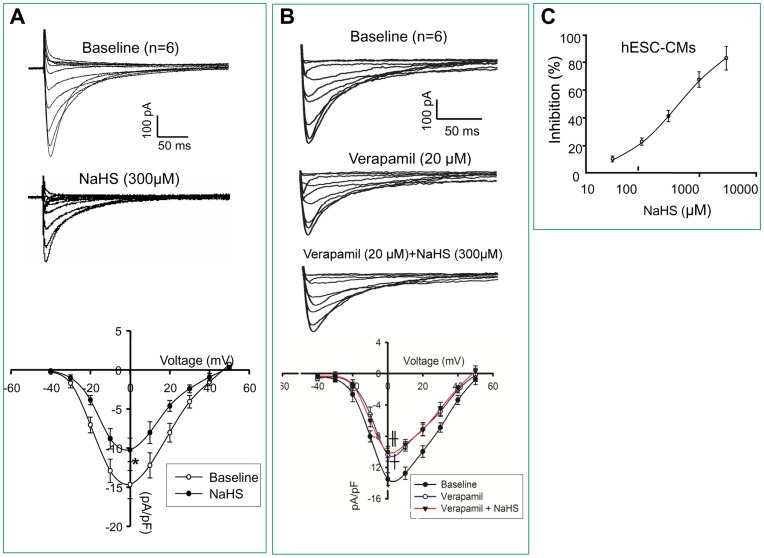
Ca^2+^ current response of hESC-CMs towards H_2_S. Representative Ca^2+^ currents were recorded on V-CMs derived from H9 hESCs. A: Traces of Ca^2+^ currents recorded before and after NaHS (300 µM) treatment. I/V curve of Ca^2+^ density was plotted. B: Ca^2+^ currents recorded at baseline, after verapamil (20 µM) treatment; and with addition of NaHS (300 µM) treatment I/V curve of Ca^2+^ density was plotted. C: The IC_50_ of the effect of NaHS on Ca^2+^ currents. *P<0.05 (NaHS treated vs. baseline). ┼p<0.05 (verapamil treated vs. baseline). ╫p<0.05 (verapamil+NaHS treated vs. baseline). Number of repeats (n) = 6.

**Figure 6:**
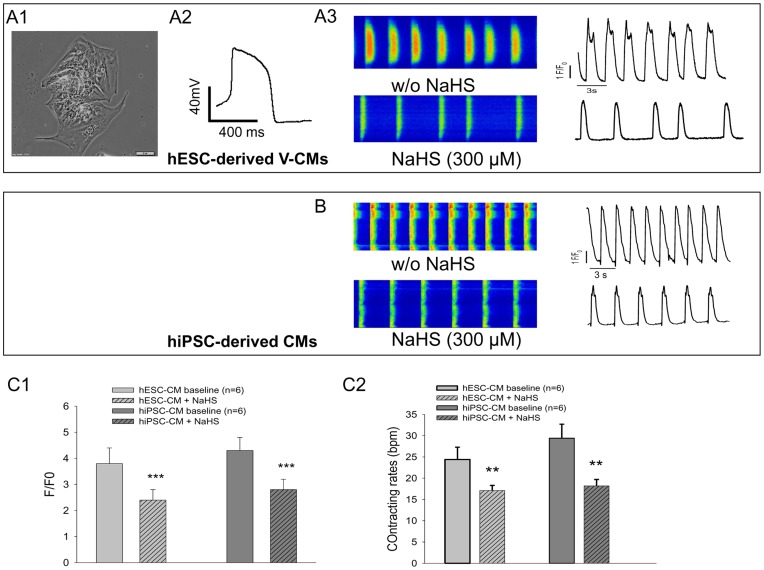
Spontaneous Ca^2+^ transients response of hESC- and hiPSC-CMs towards H_2_S. A1∼3: Light microscopy images of a cluster of H9 hESC-derived V-CMs (A1), their AP traces (A2) and the Fluo-4 loaded confocal line-scan calcium images of rhythmic spontaneous Ca^2+^ transients recorded before and after NaHS (300 µM) treatment (A3). B: Fluo-4 loaded confocal line-scan calcium images of rhythmic spontaneous Ca^2+^ transients recorded in hiPSC-CMs before and after NaHS (100 µM) treatment. C1: The time course of the ratio of fluorescence to background fluorescence (F/F0) of hESC-CM and hiPSC-CM prior to and post NaHS treatment. C2: the average contracting rates of hESC-CM and hiPSC-CM prior to and post NaHS treatment. **p<0.01, ***p<0.001 (NaHS treated vs. baseline). Number of repeats (n) = 6.

#### Measurement of outward K^+^ current

In the voltage-clamp mode, the outward K^+^ current was measured on the same V-like cardiomyocytes after AP recording. Immediately after the AP measurement, Ca^2+^ and Na^+^ currents (*I*_Ca_ and *I*_Na_) were blocked with 0.5 mM CdCl_2_ and 20 µmol/L TTX in normal Tyrode’s solution, respectively. The outward K^+^ currents were elicited by a 500-ms depolarization from holding potential of −80-mV to voltages ranging from −40 to +70 mV in 10-mV steps. Pipette solution for AP and *I*_K_ (in mM): KCl 130, MgCl_2_ 1, MgATP 3, EGTA 10, and HEPES 10, pH 7.2 with KOH. Modified extracellular solution for the AP and outward K^+^ currents (in mM): normal Tyrode’s solution plus 0.5 mM CdCl_2_ and 20 µmol/L TTX to block *I*_Ca_ and *I*_Na_, respectively.

#### Measurement of L-type Ca^2+^ currents

The H9 hESC-CM clusters contain homogeneous V-like cardiomyocytes (after consistent identification of 5 V-like cardiomyocytes in that cluster) were subsequently used for measuring of L-type Ca^2+^ currents (*I*_CaL_). The *I*_CaL_ was evoked by a 400-ms pulse to +60 mV from the holding potential of −40 mV in 10 mV increment. Pipette solution for *I*_CaL_ was prepared (in mM): CsCl 120, MgCl_2_ 3, MgATP 5, EGTA 10, HEPES 5, pH 7.2 with CsOH. A modified pipette solution for AP and *I*_CaL_ was prepared (in mM): KCl 130, MgCl_2_ 1, MgATP 3, EGTA 10, and HEPES 10, pH 7.2 with KOH. Extracellular solution for calcium current (*I*_CaL_) (in mM): TEA-Cl 140, CsCl 5.4, 4-AP 1, MgCl_2_ 1.2, CaCl_2_ 1.8, HEPES 5, glucose 10, pH 7.4 with CsOH. The recording was performed at room temperature.

#### Measurement of hyperpolarization-activated inward current (I_f_)

I_f_ current was recorded in a single hiPSC-CM. The external solution contained (in mM) NaCl 132, KCl 4, MgCl_2_ 1.2, CaCl_2_ 1.8, glucose 5, and HEPES, 10 (pH 7.4 with NaOH). The pipette solution contained (in mM) KCl 150, K_2_ATP 1, MgCl_2_ 5, and HEPES 3 (pH 7.2 with KOH). Cells were hyperpolarized from a holding potential of −40 mV to test potentials of −50 mV to −110 mV for 3000 ms to elicit currents, which were completely blocked by CsCl (2 mM).

**Figure 7:**
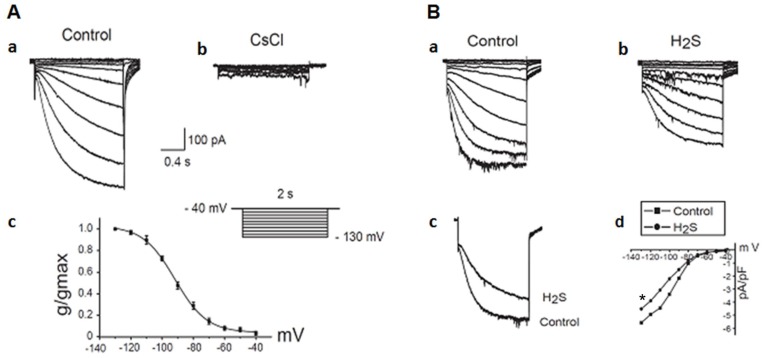
Effect of H_2_S on the hyperpolarization-activated inward current (I_f_) in hiPSC-CMs. A: Voltage- and time-dependence of the I_f_. Original I_f_ current recorded in a hiPSC-CM shows the voltage- and time- dependence (voltage clamp protocol in inset). Superimposed traces of a hiPSC-CM recorded before (Aa) and after (Ab) addition of external Cs^+^ (2 mM). (Ac) The activation curve calculated by fitting a Boltzman distribution to normalized current over a voltage range of −40 mV to −130 mV. Half-maximal activation and slope factor were −90 mV and −10.2 mV, respectively. Number of repeats (n) = 4. B: Original I_f_ traces of a hiPSC-CM before (Ba) and after (Bb) the exposure of H_2_S (100 µM). (Bc) Superimposed traces before and after exposure of H_2_S (100 µM). (Bd) Current–voltage relationship of I_f_ in the absence and presence of H_2_S (100 µM). **p<0.05 (NaHS treated vs. baseline). Number of repeats (n) = 4.

### Measurement of Confocal Ca^2+^ Transients

Calcium imaging using confocal fluorescent microscope was conducted in combination with AP recordings. Contracting clusters of homogenous H9 hESC-derived V-CMs on 3.5-cm glass bottomed dishes were identified and labeled after a consistent recording of over 5 CMs showing homogenous AP patterns of V-CM or N-CM. After 2 hours recovery in culture medium, CMs were loaded with 6 µg/mL Fluo-4 AM (Molecular Probes) for 15 min at 37°C and changed to normal Tyrode solution. Ca^2+^ transients were recorded by a LSM-710 laser scanning confocal microscope (Carl Zeiss, Inc Germany) with a × 40 oil immersion objective, numeric aperture = 1.3. Fluo-4 was excited at 488 nm using a 25 mW argon laser (with intensity attenuated to 1%). Fluorescence emission was measured at >505 nm. Images were acquired in the line (X-T)-scan mode with 512 pixels per line at a rate of 3 ms per scan. The scan line was oriented along the longitudinal axis of the cell, at pixel intervals of 0.15 µm. The axial resolution was set at 1.5 µm according to the manufacture’s specifications. In some experiments, Ca^2+^ transients were measured with the confocal microscope operating in the frame (X-Y) imaging mode. Ca^2+^ images were analyzed using a computer program written in IDL 5.4 software [12].

### Statistical Analysis

For ion current density, data were presented as mean±SEM (standard error of the mean) and reflected measurements of multiple cells. For the rest, data were expressed as mean±SD. Data were analyzed by 2-tailed Student *t* tests. P values below 0.05 were considered to indicate a statistically significant difference.

## Results

### Cardiac Differentiation of H9 hESCs and Subsequent Characterization

Contracting EBs were obtained from H9 hESCs after 15 days of cardiac differentiation with differentiation efficiency (contracting EBs vs. total EBs) ranging from 29±3% to 50±5%, which is consistent with previous reports [13]. Dissociated hESC-CMs showed sarcomeric protein expression similar to those in previous reports (**Fig. 1A**). [4], [5] Among dissociated hESC-CMs, ventricular (V)-, atrial (A)- and nodal (N)-like subtypes (**Fig. 1B1**) were identified by corresponding action potential (AP) properties which included action potential amplitude (APA), 50% and 90% of action potential duration (APD50 and APD90), and maximal rate of depolarization or maximal upstroke velocity (dV/dt_max_) (**Fig. 1B2**). However, unlike adult quiescent CMs, A- and V-like hESC-and hiPSC-CMs were capable of spontaneous contractions in culture. The proportion of 3 subtypes of CMs from H9 hESCs was similar to previous reports with V-like CMs constituting the majority subtype (60∼70%) of total cardiomyocyte populations [10], [11].

### Effect of H_2_S on hPSC-derived Cardiomyocytes

NaHS (a H_2_S donor) exerted a dose-dependent (from 100 to 1500 µM) effect on both H9 hESC- and hiPSC-CMs. **Fig. 2** shows that NaHS dose-dependently prolonged the action potential duration of a H9 hESC-derived atrial-like CMs (**Fig. 2A**) and both APD50 and ADP90 were significantly increased with approximately 50% APD prolongation (IC_50_) achieved at 300 µM (**Fig. 2B**). As the H_2_S solution contains a concentration that is approximately 33% of the original concentration of NaHS [14], 300 µM of NaHS is equivalent of ∼100 µmol/L of H_2_S. Counting on the rapid decay of H_2_S under *in vitro* condition, this concentration is close to the top limit of the physiological concentration of H_2_S which is 50∼90 µM [15], [16]. Accordingly, the subsequent experiments were performed mostly with 100 µM of NaHS and 300 µM of NaHS was used to test the maximal effects.

### Action Potential Response of hPSC-CMs towards NaHS

Exposure to NaHS evoked distinct electrical responses from the 3 different subtypes of cardiomyocytes indicated by altered action potential properties. Following NaHS exposure, beating rates were significantly reduced in V- and A-like CMs derived from H9 hESC (p<0.05) and hiPSC (p<0.05) (**Fig. 3A**). No difference was found with N-like CMs (data not shown).

APDs in the V- and A-like CMs derived from H9 hESCs and hiPSCs were significantly prolonged following NaHS exposure. There was a significant prolongation of APD in the V-like CMs derived from H9 hESCs (APD50∶504.7±17.8 ms vs. 360.3±23.5 ms, p<0.01; APD90∶612.5±19.5 ms vs. 431.2±25.0 ms, p<0.01) and hiPSCs (APD50∶519.2±15.9 ms vs. 374.7±24.6 ms, p<0.01; APD90∶593.4±17.9 ms vs. 418.2±23.6 ms, p<0.01) following exposure to NaHS (**Fig. 3B**). Similar APD prolongation was detected in A-like CMs derived from H9 hESC (APD50∶205.6±12.6 ms vs. 148.1±13.4 ms, p<0.05; APD90∶275.6±18.3 ms vs. 185.8±12.6 ms, p<0.05) and hiPSC (APD50∶182.4±6.7 ms vs. 127.8±5.5 ms, p<0.01; APD90∶266.3±7.9 ms vs. 195.6±11.5 ms, p<0.01) respectively (**Fig. 3C**). However, no statistically significant changes in APD were observed in the N-like CMs derived from H9 hESCs and hiPSCs (data not shown). In addition, no significant changes in action potential amplitude (APA), maximal upstroke velocity (dV/dt_max_) and maximum diastolic potential (MDP) were found following NaHS treatment (data not shown).

### Ion Currents Response of hESC- and hiPSC-derived CM towards H_2_S

To investigate the mechanism behind the effect of H_2_S on prolongation of APD and slowing down of the contraction rates, effects of H_2_S in altering ion currents in H9 hESC- and hiPSC- derived V-like CMs were investigated.

### Exposure to NaHS Significantly Reduced Outward Potassium Currents (*I*_K_) in H9 hESC- and hiPSC-derived CMs

Outward rectifier potassium currents (*I*_K_) including slow and rapid *I*_K_ (*I*_Ks_ and *I*_Kr_) contribute to the 2^nd^ and 3^rd^ phase of AP. With a modified protocol that facilitates the combined measurement of potassium currents with action potential, *I*_K_ was recorded in V- and A- like CMs identified by AP measurement. **Fig. 4** showed representative data recorded with V-like CMs.

It was found that exposure to NaHS (100 to 300 µM) significantly reduced total *I*_K_ in both hESC-CMs (**Fig. 4A**) and hiPSC-CMs (**Fig. 4B**). The current-voltage (*I−V*) relationships demonstrated a suppression of NaHS (300 µM) on *I*_K_ density (pA/pF) (**Fig. 4A1** and **Fig. 4B1**). However, such effect of NaHS on *I*_K_ was found to be abolished after pretreatment of cardiomyocytes with a mixture of *I*_K_ blockers containing 5 µM E-4031 (blocks *I*_Kr_) and Chromanol 293B (blocks *I*_Ks_) (**Fig. 4A2** and **Fig. 4B2**). Separately, the effects of NaHS on *I*_Ks_ and *I*_Kr_ were independently validated. **Fig. 4A3** and **Fig. 4B3** show that Chromanol 293B (a *I*_Ks_ blocker) alone suppressed *I*_K_ in both hESC-derived and iPSC-derived V-like CMs respctively. Addition of NaHS to Chromanol 293B treated cells led to further attenuation of *I*_K_ to a level close to that of NaHS treatment alone, suggesting that NaHS could additionally inhibit *I*_Kr_. Similarly, **Fig. 4A4** and **Fig. 4B4** show that E-4031 (a *I*_Kr_ blocker) suppressed *I*_K_ in both hESC-derived and hiPSC-derived V-like CMs respectively. Additional NaHS treatment resulted in a further reduction in *I*_K_ to a level close to NaHS treatment alone, suggesting that NaHS could additionally inhibit *I*_Ks_. The effects of NaHS on *I*_Ks_ and *I*_Kr_ in hESC-CMs and hiPSC-CMs were quantified and summarized in **Fig. 4A5** and **Fig. 4B5** respectively. Compared to the baseline, it was noted that NaHS registered a ∼50% suppression of *I*_K_ in hESC-CM (A1) and hiPSC-CM (B1). Such effect of NaHS was equivalent to the inhibitory effects on *I*_K_ (44.6±2.6 and 47.7±3.7) achieved by combined Chromanol 293B and E-4031 which blocked both *I*_Ks_ and *I*_Kr_ in hESC-CMs (A2) and hiPSC-CMs (B2). After subtracting the effects of NaHS from the effects of both *I*_K_ blockers, additional treatment with NaHS did not show a significant further inhibition on *I*_K_ (A2 and B2), suggesting that the effect of NaHS overlapped with those of Chromanol 293B and E-4031 blockers. Moreover, it was noted that NaHS exerted additive inhibitory effect on total *I*_K_ in the respective presence of inhibitory effects of Chromanol 293B on *I*_Ks_ (A3 and B3) and E-4031 on *I*_Kr_ (A4 and B4), suggesting that NaHS suppressed both *I*_Ks_ and *I*_Kr_ in both hESC-CMs and hiPSC-CMs. However, the effects of NaHS on A-like CMs were inconclusive due to insufficient number of cells (n <4 for each subgroup) tested. No effects of NaHS on N-like CMs were observed (data not shown).

### Exposure to NaHS Significantly Inhibited L-type Ca^2+^ Current in H9 hESC-CMs

Ca^2+^ signaling plays a crucial role in cardiac excitation-contraction (EC) coupling. L-type Ca^2+^ channel is responsible for Ca^2+^ influx triggered by the electrical signal during cardiac contraction. *I*_CaL_ contributes to the 2^nd^ phase of action potential and is more prominent in ventricular cardiomyocytes. Cardiomyocytes exposed to NaHS showed a decreased *I*_CaL_ density. **Fig. 5A** shows that the *I*_CaL_ density was significantly decreased in H9 hESC-derived V-like CMs exposed to 300 µM NaHS from −15±2.9 pA/pF to −10±3.2 pA/pF at 0 mV (p<0.05). The specificity of *I*_CaL_ was confirmed by 20 µM verapamil, a blocker of *I*_CaL_ which expectedly reduced the current substantially (p<0.05), while the addition of 300 µM NaHS in the presence of 20 µM verapamil failed to see a further reduction of *I*_CaL_, suggesting that the effect of NaHS overlapped with verapamil (**Fig. 5B**). It thus further confirmed the effect of NaHS on *I*_CaL_. Furthermore, the I-V relationships following NaHS treatment suggested a concentration-response curve of *I*_CaL_ inhibition by NaHS (**Fig. 5C**).

Moreover, confocal scanning of Fluo 4-loaded H9 hESC-derived V-like CMs (**Fig. 6A1, 6A2**) and hiPSC-CMs (with unidentified subtype) showed that exposure to NaHS (300 µM) decreased firing frequency (**Fig. 6A3** and **Fig. 6B**). Expectedly, quantitative data showed reduced amplitude of calcium transients (p<0.001) (**Fig. 6C1**) and decreased contraction rates (p<0.01) (**Fig. 6C2**) after NaHS treatment.

### Exposure to NaHS Significantly Inhibited *I*_f_ Current in hiPSC-derived CMs

Interestingly, *I*_f_ current was detected in hiPSC-derived V-like CMs which was significantly reduced by addition of specific channel blocker, CsCl (**Fig. 7A**). Similarly, exposure to NaHS (100 µM) significantly decreased *I*_f_ current in those CMs (p<0.05) (**Fig. 7Bd**).

## Discussion

Hydrogen sulfide (H_2_S) as a recently identified gaseous signalling transmitter has been known to interact with a wide range of ion channels to mediate important physiological responses. These include protecting against myocardial ischemic reperfusion injury and other cardiac protective effects through modulation of ATP-sensitive potassium current (*I*_kATP_) [17] and voltage-gated L-type calcium current (*I*_CaL_) [18]. Despite its beneficial effects, H_2_S may have implication in cardiac arrhythmias as it interacts with multiple ion channels involved in membrane action potential [7].

Outward rectifier potassium currents (*I*_K_) including slow and rapid *I*_K_ (*I*_Ks_ and *I*_Kr_) contribute to the 2^nd^ and 3^rd^ phase of AP, and both play an important role in regulating the repolarization of cardiomyocytes. Drugs affecting *I*_Ks_ and *I*_Kr_ have been associated with cardiac arrhythmia [1], [2]. Both *I*_Ks_ and *I*_Kr_ are the predominant *I*_K_ in adult human CMs [19] and their presence has been confirmed in hiPSC-CMs [10], [20], [21]. To our knowledge, the findings of inhibitory effect on delayed rectifier *I*_k_ of human CMs is the first report of such effect by H_2_S. Our data showed that H_2_S at physiological concentration could suppress both *I*_Ks_ and *I*_Kr_ currents. In addition, H_2_S was found to mediate *I*_CaL_ channel inhibition in V-like CMs derived from H9 hESCs. This is consistent with previous report that H_2_S inhibited *I*_CaL_ in rat V-like cardiomyocytes [22].

In human CMs, the outwardly rectifying potassium current *I*_Ks_ and *I*_Kr_ contribute to phase 2 and 3 repolarization of AP in both V- and A-CMs [23]. In V-CMs, the longer phase 2 of AP of membrane depolarization is contributed mainly by *I*_CaL_. In the present study, the suppressed *I*_Ks_ and *I*_Kr_ by H_2_S contributed to prolonged repolarization phase in V-like and A-like CMs with appreciable perturbation of APD. In V-like CMs, however, altered APD could be the consequence of a balanced inhibitory effect of H_2_S on delayed rectifier *I*_K_ and on *I*_CaL_. Although suppressing *I*_CaL_ is likely to shorten the APD, inhibiting outwardly rectifying potassium current in phase 2 and phase 3 by H_2_S may override such effect, resulting in an overall APD prolongation as observed in our study. In contrast to V- and A-like CMs, N-like CMs derived from H9 hESC and hiPSCs did not show significant difference in APD in response to H_2_S.

In contrast to adult CMs of which only nodal CMs are capable of spontaneous contraction, all CMs derived from hiPSCs are capable of spontaneous contraction [10] indicating their fetal-like phenotype in culture. The identification of *I*_f_ current in hiPSC-derived V-like CMs in this study further supports the immature status of those hiPSC-CMs. Nevertheless, inhibition of *I*_f_ may explain the inhibitory effect of H_2_S on the contraction rates of hiPSC-CMs observed. However, since sulfhydration of K_ATP_ by H_2_S has been attributed to its channel opening effect [24], [25], it will be interesting to study if S-sulfhydration by H_2_S had any effect on *I*_K_, *I*_CaL_ and *I*_f_ channel activities in the future. Furthermore, endogenous H_2_S mediated by cystathionine-β-synthase on the observed outcomes were not studied, though it has been shown to have effect similar to *I*_KATP_ channel opening effect of exogenously supplied NaHS in rat myocytes [26].

In conclusion, we present the first report of inhibition of both slow and rapid delayed rectifier potassium channels and hyperpolarization-activated inward current in human cardiomyocytes by H_2_S. Such effect, in combination with its inhibition of *I*_CaL_, could contribute to prolonged APD and slowed contracting rates in V-and A-like CMs that may have undesired implications in its vasodilation function in cardiac hemodynamics.
