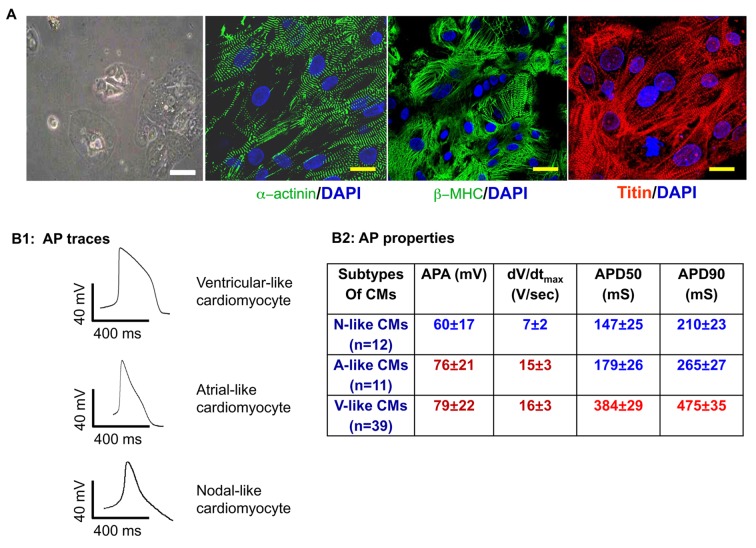# Correction: Hydrogen Sulfide Suppresses Outward Rectifier Potassium Currents in Human Pluripotent Stem Cell-Derived Cardiomyocytes

**DOI:** 10.1371/annotation/79d26ad0-169f-4440-8bee-4f483550112b

**Published:** 2013-09-04

**Authors:** Heming Wei, Guangqin Zhang, Suhua Qiu, Jun Lu, Jingwei Sheng, Manasi, Grace Tan, Philip Wong, Shu Uin Gan, Winston Shim

Due to an error in the original version of Figure 1A, a phase contrast image of human induced pluripotent stem cell-derived cardiomyocyte cluster was presented as H9 human embryonic stem cell-derived cardiomyocyte cluster. The authors apologize for this error and are supplying a corrected Figure 1A that displays H9 human embryonic stem cell-derived cardiomyocytes.

Figure: 

**Figure:**